# Classifying evolutionary forces in language change using neural networks

**DOI:** 10.1017/ehs.2020.52

**Published:** 2020-10-16

**Authors:** Folgert Karsdorp, Enrique Manjavacas, Lauren Fonteyn, Mike Kestemont

**Affiliations:** 1 Royal Netherlands Academy of Arts and Sciences, Meertens Institute, Amsterdam, The Netherlands; 2 Department of Literature, University of Antwerp, Antwerp, Belgium; 3 Leiden University Centre for Linguistics, Leiden University, Leiden, The Netherlands

**Keywords:** cultural evolution, language change, drift, selection, neural networks

## Abstract

A fundamental problem in research into language and cultural change is the difficulty of distinguishing processes of stochastic drift (also known as neutral evolution) from processes that are subject to selection pressures. In this article, we describe a new technique based on deep neural networks, in which we reformulate the detection of evolutionary forces in cultural change as a binary classification task. Using residual networks for time series trained on artificially generated samples of cultural change, we demonstrate that this technique is able to efficiently, accurately and consistently learn which aspects of the time series are distinctive for drift and selection, respectively. We compare the model with a recently proposed statistical test, the Frequency Increment Test, and show that the neural time series classification system provides a possible solution to some of the key problems associated with this test.

## Graphical abstract

**Figure:**
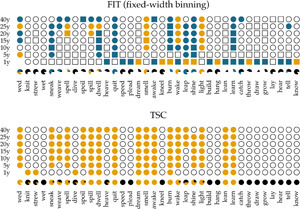

**Media summary:** We develop a new method based on neural networks to distinguish between cultural selection and drift.

## 1. Introduction

To study the mechanisms underlying cultural change, detailed information is needed about the complex mix of, for example, cognitive, social, and memory-based biases of individuals that bring about a certain change. However, for most real-world examples of cultural change, information at the level of individuals is not available, thus forcing us to resort to (shifts in) frequency distributions at the population level. A central challenge in research into cultural change is, therefore, to develop methodologies and techniques that can infer biases active at the level of individuals from signatures in population-level statistics (Acerbi & Bentley, 2014; Kandler & Powell, 2015; Kandler & Shennan, 2013; Mesoudi & Lycett, 2009). Recently, various inference techniques have been proposed, in which observed, real-world population-level statistics of cultural change are compared and contrasted with the outcomes of theoretical simulation models. By investigating divergences between simulated and real-world frequency distributions (Bentley et al., 2004, 2007; Hahn & Bentley, 2003; Herzog et al., 2004; Ruck et al., 2017) or turnover rates (Acerbi & Bentley, 2014; Youngblood, 2019), or applying likelihood-free inference techniques (Carrignon et al., 2019; Crema et al., 2014, 2016; Kandler & Powell, 2015; Kandler & Shennan, 2013, 2015; Lachlan et al., 2018), arguments for the presence of individual-level biases underlying cultural change have been made, such as conformity bias in bird song (Lachlan et al., 2018) and music sampling traditions (Youngblood, 2019), or anti-conformity bias in archaeological pottery data (Crema et al., 2016). Knowledge of such biases operating at the individual level is crucial to better understand how they ‘can affect the populational profile of a collection of ideas, skills, beliefs, attitudes, and so forth’ (Lewens, 2015, p. 57, and see Boyd & Richerson, 1985; Cavalli-Sforza & Feldman, 1981; Richerson & Boyd, 2005).

Despite these advances, it remains challenging to single out specific individual-level processes underlying cultural change with sufficiently high certainty. As such, it has been proposed to shift attention to the *exclusion* of certain mechanisms that are unlikely to have produced the observed data (Kandler et al., 2017). In a similar vein, it has been proposed to first establish whether there is evidence for selection in the first place, before examining specific selection processes and individual-level biases (Brantingham & Perreault, 2010; Feder et al., 2014; Lycett, 2008; Newberry et al., 2017; Zhai et al., 2009). A recent proposal (Newberry et al., 2017) is to employ the ‘Frequency Increment Test’ (FIT), which is borrowed from population genetics (Feder et al., 2014). The FIT provides an elegant tool to test for the presence of directed selection in processes of, for example, language change, against a null model of stochastic drift (i.e. unbiased selection). The FIT has been used to systematically quantify the role of biased selection and unbiased, neutral change in a number of grammatical changes in English. The results highlight the importance of selectional forces in language change, but at the same time they emphasize the often underappreciated role of stochasticity in language change (Baxter et al., 2006; Bentley et al., 2011; Kauhanen, 2017; Reali & Griffiths, 2010; Ruck et al., 2017) – and, by extension, cultural change in general (Carrignon et al., 2019; Karsdorp & Van den Bosch, 2016).

While promising, a systematic, critical assessment of the applicability of the FIT to linguistic data demonstrates that the statistical power of the FIT (i.e. the probability of the FIT correctly rejecting the null model of stochastic change) is sensitive to a number of factors (Karjus et al., 2020). First, when working with linguistic or cultural data, researchers are often confronted with sparse and incomplete data, both in space and in time. This sparsity forces researchers to group (i.e. ‘bin’) linguistic variants within a specific geographical region or time period. It is shown that the number of temporal segments severely impacts the statistical power of the FIT (Karjus et al., 2020). Most importantly, the number of false positives increases when fewer bins are available, both when selection strength is high and when selection strength is absent (i.e. with stochastic drift). Second, since the statistical test underlying the FIT is a one-sample *t*-test (see below), the assumption of normality must be accounted for. However, in linguistic and cultural time series of frequency increments, the normality assumption is often violated, thus rendering the FIT results uninterpretable. Finally, the statistical power of the FIT is generally weak when selection coefficients are either too low or too high. In case they are too low, the generated time series become indistinguishable from those produced by stochastic drift. If, on the other hand, selection coefficients are too strong and few data points are available (e.g. owing to the binning strategy applied), changes might take place too fast to be noticed by the FIT (Feder et al., 2014; Karjus et al., 2020).

In this article we reformulate the problem of detecting evolutionary forces in cultural change as a time series classification problem. The method we propose employs Residual Networks (Fawaz et al., 2019; Wang et al., 2016), trained on time series simulated with the Wright–Fisher model (Ewens, 2012). The neural networks are able to efficiently and accurately learn which aspects of the time series are relevant to distinguish stochastic drift from changes subject to selection pressure. We critically compare and contrast the performance and behaviour of the neural classifier with that of the FIT, and show how it solves a number of problems of the latter:

1. First, the neural networks are barely affected by varying numbers of temporal segments, thus effectively solving the aforementioned binning problem.
2. Second, the neural networks do not assume a particular distribution underlying the data, which increases their applicability to time series with non-normally distributed frequency increments, and for example, time series following sigmoid S-curves often observed in language and cultural change (Acerbi et al., 2016; Blythe & Croft, 2012; Denison, 2003; Smaldino et al., 2018).
3. Third and finally, we show that the neural networks are affected less by distortions of the time series compared with the FIT, making the method more applicable to the noisy, sparse and incomplete data that we often find in historical collections of cultural data.

After a critical assessment of the behaviour and performance of the proposed method, we apply the neural network to a real-world data set, and discuss its predictions in relation to those of the FIT.

## 2. Methods

### 2.1. The Frequency Increment Test

The FIT (Feder et al., 2014) is based on the key idea that statistics at the population level have certain characteristics that can be traced to processes or behaviour at the individual level. For example, it is hypothesized that processes subject to selection forces look different from processes driven by stochastic drift, and that these processes leave their signature in the observed statistics. The statistics studied here are time series consisting of ordered sets of relative frequencies of cultural variants. For a time series of length *T*, we calculate at each time point *t*_*i*_ the relative frequency *f*(*t*_*i*_) of the variants of a cultural trait. Each time series *X*_*i*_ can thus be described as a univariate series *X*_*i*_ = [*f*(*t*_1_), *f*(*t*_2_), …, *f*(*t*_*T*_)].

The FIT operates on these time series by rescaling them into ordered sets of frequency increments *Q*:1
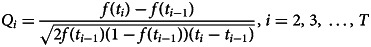


where *f*(*t*_*i*_) represents the relative frequency of a cultural variant at the current time step *t*_*i*_, and *f*(*t*_*i*−1_) that in the previous one. The reason for this rescaling is that *Q* is approximately normally distributed under stochastic drift, with a mean of zero. In contrast, when selection pressures are present, the distribution is also normally distributed, but with a non-zero mean. Rescaling the data in this way allows us to employ a classical *t*-test to investigate whether the frequency shifts in a time series are subject to drift (*H*_0_) – in which case the mean frequency increment does not deviate significantly from zero – or to selection (*H*_1_) – in which case the mean increment deviates significantly from zero. The null hypothesis of unbiased selection is rejected if the two-sided *p*-value of the *t*-test is below some threshold *α*. In this study, we set *α* to 0.05. As the FIT assumes frequency increments to be normally distributed, we need to test this assumption. To this end, we follow prior work and perform a Shapiro–Wilk test, with a *p*-value threshold of 0.1 (Karjus et al., 2020).

### 2.2. Time series classification

#### 2.2.1. A machine learning approach

As an alternative to the FIT, we propose to conceptualize the task of detecting evolutionary forces in language and cultural change as a binary time series classification (TSC) task. In this respect, our methodology is borrowed from the field of supervised classification research in machine learning (Sen et al., 2020), which is concerned with the development of computational models that can be trained on example data to learn how to automatically assign (unseen) instances from a particular domain into a set of (mutually exclusive) categories – such as a positive or negative class in the case of a binary classification setup like ours. More specifically, we resort to a sequence classifier, which will map an input in the form of a time series vector to one of two category labels (i.e. the absence or presence of selection pressure in a time series). Formally, given a data set *D* consisting of *N* pairs of time series *X*_*i*_ and corresponding labels *Y*_*i*_ ∈ 0, 1, i.e. *D* = (*X*_1_, *Y*_1_), (*X*_2_, *Y*_2_), …, (*X*_*N*_, *Y*_*N*_), the task of TSC is to learn a mapping function for the input series to the output labels. *Y*_*i*_ = 1 when *X*_*i*_ was produced under selection forces, and *Y*_*i*_ = 0 otherwise.

For this purpose, we use deep neural networks, a broadly applicable learning framework which has recently gained much popularity (LeCun et al., 2015; Schmidhuber, 2015). An important advantage of neural classifiers is that no advanced feature engineering is needed on the researcher's side in order to present the data to the model in an optimal format: neural networks are designed to independently learn which characteristics in the input are most useful to solve a particular classification task. A conventional deep neural network takes the form of what is known as a multilayer perceptron (LeCun et al., 2015; Schmidhuber, 2015), a networked structure through which information can be propagated: the architecture feeds an input vector, representing an instance (such as a time series), through a stack of layers that consecutively transform the input through multiplying it with a weight matrix, followed by a non-linear activation function (such as the sigmoid), to bound the output. The more intermediate or ‘hidden’ layers such a ‘deep’ network has, the more modelling capacity it provides to fit the data. In the case of a network for binary classification, the last layer will transform the output of the penultimate layer into a single score that can be interpreted as the probability of the positive class (e.g. the presence of selective force in the series of trait frequencies). To bound the output score to a suitable range, a squashing function can be applied, such as the logistic function. Nowadays, neural networks are trained with a procedure known as stochastic gradient descent, where each layer's weight matrix is progressively optimized in light of an objective function or criterion that monitors the network's loss or how strongly its predictions diverge from the ground truth in the training data. By fine-tuning these weights in multiple iterations over the available training data, the classification performance of the network gradually improves.

#### 2.2.2. Residual networks

More specifically, we employ residual networks (He et al., 2016), which have been shown to act as a strong baseline, achieving high quality and efficiency on a rich variety of time series classification tasks (Fawaz et al., 2019; Wang et al., 2016). A residual neural network is characterized by the addition of so-called ‘skip-connections’ that link the output of a layer with the output of another layer more than one level ahead. The introduction of residual blocks has been crucial to enable the training of deeper networks, resulting in increasingly strong performance (He et al., 2016; Srivastava et al., 2015). The network architecture underlying the present study consists of three residual blocks. Instead of plain linear transformations followed by a non-linear function, each residual block is composed of weights that are ‘convolved’ with the input vector (LeCun et al., 1998; Szegedy et al., 2015). Each of these convolutional weights (typically known as a convolutional filters or kernels) is slid over the input values, generating a windowed feature vector for consecutive segments of the timeseries.

The concept of convolutional filters was originally developed in computer vision (LeCun et al., 1998) to enable the detection of meaningful, local, spatial patterns regardless of their exact position in a time series. For the present study, each residual block consists of three convolutional blocks that have 64 filters of size 8, 128 of size 5 and 128 of size 3. The outputs of all convolutional filters are passed through the non-linear rectified linear unit activation function (Nair & Hinton, 2010) and concatenated into an output matrix of dimensionality proportional to the input size and the number of filters. The output matrix of the last residual block is transformed into a single vector by averaging over all the units (i.e. global average pooling). Finally, this vector is passed into the last layer which outputs a scalar that is transformed into a probability with the logistic function. For more information and further details about the mathematical definition of the architecture and training details, see the original proposal (Wang et al., 2016) and the Supplementary Materials accompanying this paper (Karsdorp et al., 2020).

#### 2.2.3. Generation of training data

A supervised classification system requires labelled examples or training material in order to optimize its weights (which are initialized randomly). However, no extensive data sets are available of linguistic data, in which the development of certain cultural traits has been annotated for particular evolutionary forces. The solution to this problem is to simulate artificial training data. We employ a simple Wright–Fisher model (Ewens, 2012) to simulate a sufficient amount of time series representing frequency changes over time. The model assumes a population of constant size *N* and discrete, non-overlapping generations. We define *z*(*t*_*i*_) as the number of times some cultural variant *A* occurs in generation *t*_*i*_, and *f*(*t*_*i*_) as the relative frequency of that variant. Under a neutral, stochastic drift model, the occurrence count of *A* in generation *t*_*i*+1_ is binomially distributed:2
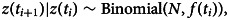


where Binomial(*N*, *f*(*t*_*i*_)) is a binomial distribution with *N* trials (i.e. for each individual in the population) and a probability of success *p* = *f*(*t*_*i*_). A more general formulation, which allows for selection pressures on the cultural variants, is the following:3
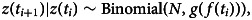


where *g* is a function with which the sampling probability of a cultural variant is altered (Tataru et al., 2016). With *β* representing the bias towards the selection of one of the variants, we define the following linear evolutionary pressure function to alter the sampling probability:4
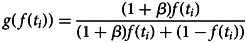


Note that when *β* = 0, the model reduces to stochastic drift. With this model we simulate time series with *T* = 200 generations, a population of *N* = 1000 individuals, and varying selection coefficients (see below for more information about how the data was simulated during training). Starting frequencies at *t*_*i*_ = 0 are sampled from a uniform distribution 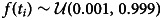.

#### 2.2.4. Data distortion

For the time series classifier to be effective, an important challenge is to simulate data that are representative of real-world time series. After all, while neural networks are likely to generalize beyond data samples seen during training, data samples that are too distant or different from the training material may hurt the performance of the models. This, of course, is a problem common to every supervised system, given its dependence on the amount and diversity of available training material. However, since the training material is simulated, we can apply certain data distortion strategies to make the data more realistic (Fawaz et al., 2018; Le Guennec et al., 2016). As a proof of concept, we propose the following two data distortion strategies:

1. *Frequency distortion* **–** it is rare for time series of cultural data to be complete. Usually we have to deal with messy, battered data, that for whatever reason are incomplete, contaminated or otherwise distorted. As a simple, albeit somewhat naive way to approximate such real-world aberrations, we propose to augment the relative frequencies ***f***(***t***_***i***_) of the Wright–Fisher model with an error term ***δ***. For each time step ***i*** = 1, 2, …, ***T***, we sample an error term from a normal distribution with zero mean and variance ***σ***:
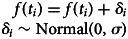
The augmented frequencies are subsequently truncated to the interval [0, 1].
2. *Varying temporal segments –* As a second strategy to mimic real-world time series distortions, we propose grouping the time series into varying numbers of temporal segments. This data augmentation strategy is again motivated by the fact that data points are often missing in cultural data, resulting in time series with either high-frequency fluctuations owing to the few data points per time step, or time steps with no data at all. To circumvent this issue, a common strategy in cultural analyses is to group the data points of a time series into a reduced number of temporal segments, or bins. We randomly sample a number of bins in the range [4, ***T***] and group the simulated time series accordingly before computing the relative frequencies in each new time step.

#### 2.2.5. Training procedure

We train the TSC using mini-batches of simulated time series. In each training epoch, 50,000 time series are generated, which, using a batch size of 500, are split into 100 mini-batches. Each time series in a mini-batch, as described above, is then simulated with a selection coefficient *β* in the range [0, 1]. Subsequently, it is binned into a randomly sampled number of temporal segments and the bin values are distorted as described above. To ensure that, after varying the number of temporal segments, all time series in a mini-batch have the same length, we apply zero-padding, in which the time series are extended with zeros, as necessary. Positive selection coefficients, *β* > 0, are sampled from a log-uniform distribution, which ensures that we obtain many samples with low selection pressure. These samples are the most difficult ones to distinguish from stochastic drift (Karjus et al., 2020), and as such, help the network in reaching more efficient and faster convergence. Importantly, the ratio of positive and negative instances in the data are kept balanced in the generated data (i.e. 50–50%). We employ the Adam optimizer (Kingma & Ba, 2015) with a small learning rate of 6×10^−5^. The loss function we aim to optimize is the binary cross-entropy loss.

For each epoch in the optimization regime, a new set of training data is generated. We monitor the network's performance after each epoch on a held-out development set (that is generated analogously to the training data, but only once at the start of the regime). Finally, the training procedure is halted after no improvement in the loss on the development data has been observed for five, consecutive epochs. For further details, we refer to the Supplementary Materials accompanying this paper.

## 3. Results

### 3.1. Critical parameter analysis

**Figure 1.**
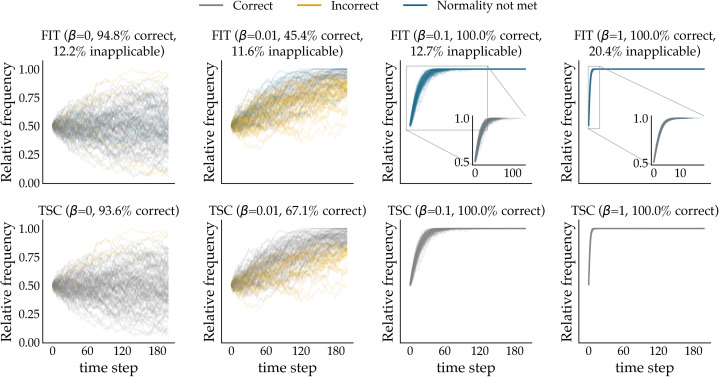
Time series generated with the Wright–Fisher model with increasing selection coefficients *β*. For each selection strength value *β*, we simulated 1000 time series of 200 generations (100 shown). Starting frequency values were set to 0.5. The top row displays the results for the FIT, and the bottom row shows the results of applying the time series classifier. Grey lines indicate correct classifications, yellow lines mark incorrect classifications and blue lines indicate inconclusive cases for which the normality assumption is not met. The accuracy scores for the FIT are computed by excluding non-normally distributed time series. The percentage of time series for which the test is inapplicable is given.

We first validate the time series classifier without varying the number of temporal segments (*T* = 200). Figure 1 displays time series generated with the Wright–Fisher model with increasing selection coefficients *β*. All simulations were run for 200 generations (see the Methods section for more details about the parameter settings). The top row shows the results for the FIT. For each time series, we calculate the FIT *p*-value, and classify time series with a *p*-value higher than 0.05 as examples of stochastic drift. Correct classifications are coloured grey, incorrect ones are marked with a yellow colour, and time series with non-normally distributed frequency increments are coloured blue. Each subplot provides a classification accuracy score, which was computed based on 1000 simulations. The accuracy score for the FIT is computed by excluding non-normally distributed time series. The plots provide the percentage of cases in which the FIT was not applicable owing to normality violations. In the bottom row, we present the results of the neural network classifier, with the same colouring for correct and incorrect classifications.

With an accuracy score of approximately 94.8% when selection is absent (i.e. *β* = 0), the FIT has a small false positive rate of 5.2%. This result aligns with prior analyses that reported a value of around 5% (Karjus et al., 2020). The time series classifier has a slightly higher false positive rate (6.4%), corresponding to an accuracy score of about 93.6%. Note, however, that the FIT is not applicable for approximately 120 of the 1000 generated time series, since they fail the normality test. As was shown in previous analyses (Feder et al., 2014; Karjus et al., 2020), the statistical power of the FIT is low with very small selection coefficients. At *β* = 0.001, most simulated time series are virtually indistinguishable from stochastic drift (6% accurate and 8.5% inapplicable). This problem also plays a role for the neural classifier, but to a slightly lesser extent (10.2% accurate). Once the selection strength becomes more pronounced (i.e. *β* ≥ 0.01), both the FIT and the classifier are able to discriminate between time series subject to drift and selection more accurately. However, with *β* ≥ 0.01, the normality assumption required for the FIT's underlying *t*-test is often not met, because the stronger selection coefficients can quickly lead to absorption events (i.e. frequency changes to 1 or 0 followed by a series without change). Strictly speaking, we should treat these non-normal time series (cf. the many blue lines in the third and fourth columns) either as misclassifications, or as cases where the FIT is simply not applicable. To remedy this situation, we truncate all values after the absorption events (cf. the inset graphs in the third and fourth columns), and subsequently compute the accuracy scores for these truncated time series. With an accuracy score of 100% for *β* = 0.1 and *β* = 1, the FIT is able to accurately discriminate between drift and selection. However, the number of cases in which the test cannot be applied increases sharply with higher values of selection pressure (12.7% for *β* = 0.1 and 20.4% for *β* = 1). Not being affected by the normality assumption, the time series classifier requires no post-hoc truncation, is applicable to all time series, and accurately predicts all time series generated with *β* ≥ 0.01 to be subject to selection.

Without binning, the two methods yield comparable performance. However, when binning is applied, marked performance differences arise. The differences in performance are revealed primarily in the false-negative rate (where time series are incorrectly classified as examples of stochastic drift), while both methods display similar false-positive rates (where selection pressures are erroneously assumed). We first focus on the differences in the false-negative rate, and subsequently address the false positives.

**Figure 2.**
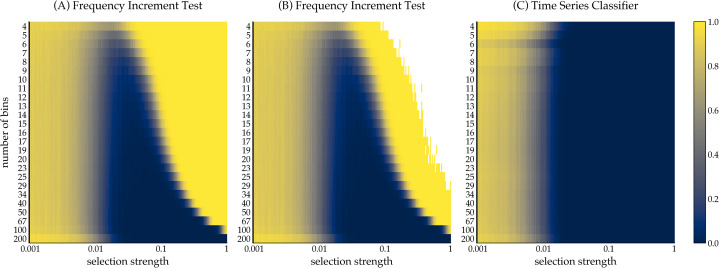
Interaction between selection coefficients and number of bins. For each unique combination of selection coefficient and number of bins, we simulate 1000 time series using the Wright–Fisher model, and compute their mean error rate. Simulations were run for 200 generations. The simulated time series were subsequently binned according to the specified number of bins. Subplot (A) displays the FIT results. Subplot (B) plots the same results but masking (a) all samples violating the normality assumption and (b) all samples with too few data points after adjusting for absorption events (white colour). The results for the time series classifier are shown in subplot (C). The colour bar at the far right of the plot functions as a legend of the error rate values.

In Figure 2, we show the interaction between different selection coefficients and varying numbers of binning (for the same, unaltered model from the previous section). The *y*-axis represents the number of bins used to group the data points, ranging from 4 to 200 bins (which, being equal to the number of generations, amounts to no binning). We simulated 1000 time series for 200 generations for each combination of selection coefficient and number of bins. The left subplot (A) shows the mean error rate per parameter combination for the FIT. The results for the FIT are largely in line with those from earlier research (Karjus et al., 2020). First, as we discussed before, the statistical power of FIT is generally lower when there is little selection pressure. In this context, binning does not seem to play a role, either negatively or positively. However, the impact of binning on the performance of the FIT becomes more articulated as the selection strength becomes stronger. With higher selection coefficients and increasingly fewer bins, the statistical power of the FIT degrades rapidly. Additionally, the normality assumption of the FIT is increasingly violated with stronger selection values and fewer bins as shown in the middle subplot (B) in which (a) non-normal samples and (b) samples with too few data points after adjusting for absorption events are left out. This negative impact of binning on the FIT contrasts sharply with the insensitivity of the time series classifier to varying temporal segments. Indeed, the right subplot (C) makes it abundantly clear that the performance of the classifier is primarily influenced by selection strength, but not by the chosen number of bins.

**Figure 3.**
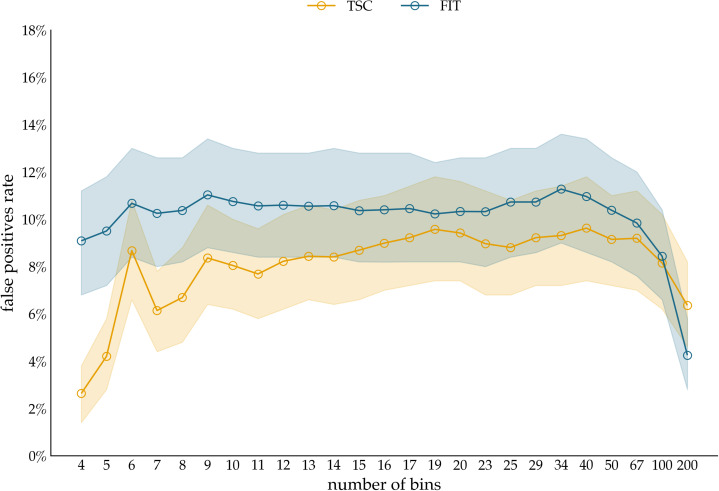
The impact of binning on the false-positive rate of the FIT and the time series classifier. With *β* set to 0 (i.e. stochastic drift), we simulate 1000 time series for each number of bins using the Wright–Fisher model. Simulations were run for 200 generations. The plot shows the mean error-rate for each bin number (solid lines), as well as the 95% confidence interval (shaded area) which was computed using a bootstrap procedure.

This relative insensitivity to binning also manifests itself when selection pressure is completely absent, that is, in the context of stochastic, unbiased selection. This is visualized in Figure 3, which draws the mean false positive rate at increasing numbers of temporal segments. The false-positive rate of the TSC is only mildly affected by varying numbers of bins. On average, and leaving out samples with non-normally distributed frequency increments, the false-positive rate of the FIT is slightly higher than the TSC, with mean false-positive rates of 10.1 and 8.1%, respectively. Thus, the analyses of the false-negative and false-positive rates seem to suggest that the neural time series classifier is robust to binning variation.

### 3.2. Application to real-world data

In the previous section, we compared the performance of FIT and TSC based on simulated data. The analysis showed that the TSC is less sensitive to variation in binning than the FIT, and furthermore that the TSC is unaffected by specific assumptions about the underlying distributions of the time series (such as the normality assumption of frequency increments). In this section, we continue our evaluation and comparison of the systems by applying them to the historical process of (ir)regularization of English past-tense verbs, which is the main grammatical change described in the original proposal to apply the FIT to language change (Newberry et al., 2017). The case of verb (ir)regularization was also recently thoroughly reviewed to assess the applicability of FIT to language change (Karjus et al., 2020). In a nutshell, the linguistic change under scrutiny simultaneously concerns the rise of ‘regular’ past tense forms such as *helped* at the expense of ‘irregular’ forms such as *holp*, as well as the rise of irregular past tense forms such as *wore* and *lit* at the expense of the (initially dominant) regular forms *weared* and *lighted*. Both changes, regardless of their direction (towards a regular or irregular form), are commonly described in the linguistic literature as cases of analogical change (Fertig, 2013).

**Figure 4.**
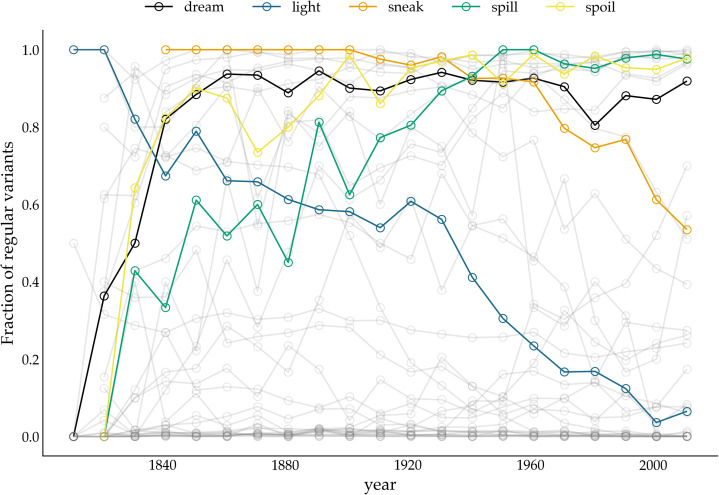
Relative frequency of past tense variants over time. The *y-*axis displays the fraction of regular variants of a particular verb. The variants of five verbs (*dreamt–dreamed*, *lit–lighted*, *snuck–sneaked*, *spillt–spilled*, *spoilt–spoiled*) are highlighted.

We apply the two methods, FIT and TSC, to a previously examined set of 36 verbs, which either regularized or irregularized in the Late Modern English period. As in the previous accounts that addressed detecting patterns of drift and selection by considering verb (ir)regularization in Late Modern (American) English (Karjus et al., 2020; Newberry et al., 2017), we extract all past-tense occurrences of each of these 36 verbs from the Corpus of Historical American English, which covers a time period between 1810 and 2009 (Davies, 2010). Subsequently, we calculate how often the regular instances occur in relation to the irregular instances per year (for more information about data (pre-)processing, see Newberry et al., 2017; Karjus et al., 2020). To allow comparison with results from previous research (Karjus et al., 2020), we apply two binning strategies for the FIT. The first is a commonly used fixed-width binning strategy, in which all occurrences of a verb within equally sized time windows are collected, and their counts subsequently summed. We group the verb occurrences into time windows of 1, 5, 10, 15, 20, 25 and 40 years (Figure 4 displays some example time series with the time window set to 10 years). However, a potential problem with this binning strategy is that the verb data are not distributed uniformly in time, which violates the FIT's requirement that each measurement has about the same variance (homoscedasticity), and causes the normality test to fail frequently. To remedy this issue, Newberry et al. (2017) and Karjus et al. (2020) apply a variable-width binning strategy, in which time series are grouped into a number of quantile bins, *n*(*b*), consisting of roughly the same number of tokens. The number of variable-width bins *n*(*b*) is computed by taking the log of the total number of past-tense tokens *v* of a particular verb, ⌈ln(*v*)⌉ (see Newberry et al., 2017; Karjus et al., 2020 for more information). To further control the number of variable-width bins, Karjus et al. (2020) experiment with a constant *c*, ⌈*c* ln(*v*)⌉, which we also adopt in the analyses below. Since homoscedasticity is not required for the TSC, we can resort to the fixed-width binning strategy here.

**Figure 5.**
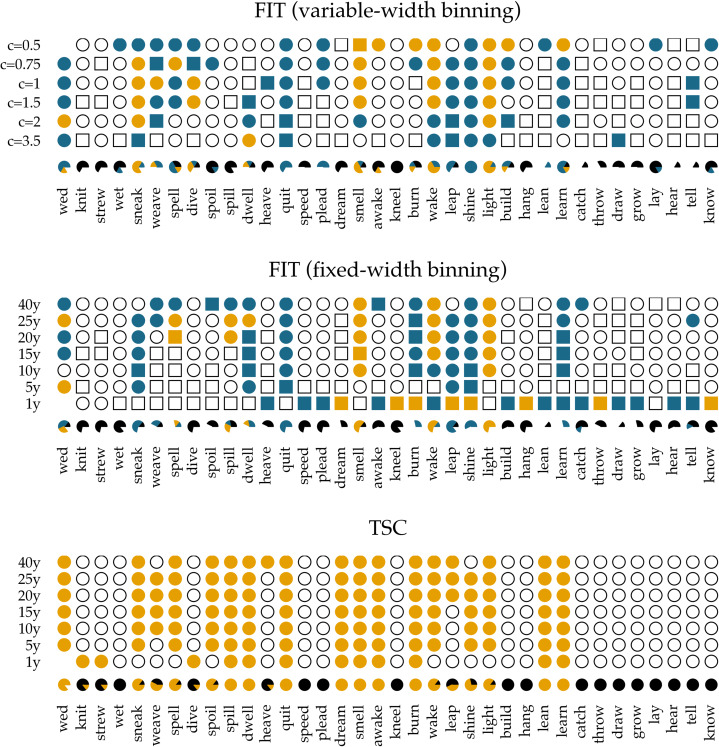
Results of applying the FIT and the TSC to the verb time series. The upper panel displays the results for the FIT with a variable-width binning strategy, the middle the results for a fixed-width binning strategy and the bottom panel those for the TSC. Circles indicate that the normality assumption of the *t*-test is met (according to a Shapiro–Wilk test with a threshold of *p* ≥ 0.1), while squares indicate that it is not met (*p* < 0.1). The colouring of the circles or squares corresponds to the results of the FIT. Unfilled items correspond to a FIT *p*-values of ≥0.2. Blue items have a *p*-values of <0.2, and yellow items have a *p*-value <0.05. The bottom panel shows the results for the TSC. Items with a probability greater than 0.5 to be generated through selection are coloured yellow; the others are unfilled. The bottom rows underneath each panel display little pie charts, which provide information about the classifying consistency of the two systems across the different binning strategies. The colour black marks neutral, stochastic drift (with a FIT *p*-value ≥0.2 and a TSC probability value ≤0.5). Blue is reserved for the FIT results and corresponds to time series with a *p*-value smaller than 0.2. Yellow is used for time series with a corresponding FIT *p*-value of <0.05, as well as for series with a TSC probability value >0.5. Finally, white is used for time series that violate the normality assumption.

In Figure 5, we show the results of applying the FIT and the TSC to the time series of past-tense verbs. The upper panel shows the results of the FIT with a variable-width binning strategy using different constants *c*. The middle panel presents the results for the fixed-width binning for the seven different binning strategies of 1, 5, 10, 15, 20, 25 and 40 years. The top and middle panels are exact reproductions of the results shown in Figure 1 of Karjus et al. (2020). The circles represent time series that meet the normality assumption of the frequency increments. Squares, on the other hand, indicate that the normality assumption is violated. The colour fill of the circles and squares corresponds to the *p*-values returned by the FIT. Unfilled items correspond to a FIT *p*-value of >0.2, which should indicate that these time series are subject to stochastic drift. Blue-coloured circles and squares correspond to a *p*-value of <0.2, and if an item is coloured yellow, the FIT *p*-value is <0.05. In both cases, this indicates that the time series were produced under some selection pressure. Finally, the consistency of the predictions of the FIT across the different binning strategies is summarized in the pie charts underneath each panel. In these charts, black parts represent the fraction of time series classified as stochastic drift, whereas blue and yellow parts represent time series undergoing selection with a *p*-value of <0.2 and <0.05, respectively. Time series violating the normality assumption are masked with the colour white.

In accordance with Newberry et al. (2017), we find that with both variable-width (top panel) and fixed-width (middle panel) binning, the frequency increments of several verbs (e.g. *smell*, *light*, *spell* and *wake*) deviate significantly from zero, allowing us to reject the null-hypothesis of stochastic drift with a *p*-value of <0.05. Relaxing the threshold of *p* < 0.05 to 0.2, the set of verbs flagged as undergoing selection increases slightly, adding verbs such as *quit*, *learn* and *sneak*. In addition, there is a large number of verbs for which no selection signal is detected by FIT: any frequency changes that verbs such as *know*, *draw* and *dream* have undergone are attributed to processes of stochastic drift. However, whether FIT detects a selection signal strongly depends on the chosen binning strategy (Karjus et al., 2020). Using a variable-width binning strategy, 26 of the 36 verbs are seen as examples of both selection and neutral evolution, depending on the number of quantile bins; employing a fixed-width binning, only five verbs are unambiguously classified across different binnings (i.e. *knit*, *strew*, *wet*, *dive* and *lay*). Moreover, the pie charts show that the prediction consistency across bins decreases even further when the cases that violate the normality assumption of the frequency increments are left out. In extreme cases such as *draw*, *lean*, *dream*, *burn* and *spell*, this drastically reduces the number of usable test outcomes (both using the variable-width and the fixed-width binning strategy), thus limiting the conclusion we can draw.

The bottom panel presents the results for the TSC. Since the normality assumption of the frequency increments does not play a role for the TSC, only circles are displayed. The colours correspond to the probabilities produced by the TSC, with unfilled circles indicating stochastic drift (with a probability <0.5), and filled yellow circles indicating a selection process underlying the time series (with a probability >0.5). Two important observations can be made when studying the results of the TSC. First, the TSC's predictions appear more consistent than those of the FIT, and are less subject to variation owing to the chosen binning strategy. The eight most frequent verbs (from *know* to *catch*) are all consistently classified as examples of stochastic drift. We find this consistency in 15 more verbs, with verbs such as *learn*, *lean*, *burn* and *dream* as examples of selection, and, for instance, *hang*, *build*, *plead* and *speed* as examples of drift. In total, 24 out of 36 verbs are consistently classified as either selection or drift. This contrasts sharply with the small number of consistently classified verbs by the FIT. In addition to differences in predictive consistency, there are also differences in which verbs are considered examples of selection or drift. Those differences consist only of verbs for which the FIT could not detect a selection signal, while the TSC identifies them as examples subject to selection. In other words, if FIT designates a verb as undergoing some selection pressure, then the TSC does too, but not vice versa. Interestingly, these are often cases where the frequency increments are not normally distributed, for example, the verb *dream* is (predominantly) classified as drift by the FIT, while the TSC marks it as subject to selection. The sharp rising frequency curve of *dream* in Figure 4 is probably the reason why the frequency increments are not normally distributed: as noted before (Karjus et al., 2020), the FIT does not cope well with such S-curve-like increases, with non-normally distributed frequency increments and high selection coefficients. Similarly, we can explain the differences between FIT and TSC for verbs such as *spill*, *spoil* and *spell*, which are also characterized by rapidly increasing, non-normally distributed frequency increments. In conclusion, compared with previous research based on the FIT (Newberry et al., 2017), the TSC attributes more verbs to selectional processes. However, a significant group of verbs that do not contain a selection signal according to both FIT and TSC remain. Thus, we should not interpret the results as an invalidation but rather as a refinement of the role of stochasticity in language change.

## 4. Discussion

In this article, we have described a new system we developed to detect evolutionary processes underlying linguistic and cultural change in general. The system formulates the problem of detecting evolutionary forces as a classification task, in which residual networks are trained on artificially generated time series of cultural change. We compared the performance of this neural TSC with the FIT (Newberry et al., 2017), which has been thoroughly evaluated as a tool to test for the presence or absence of selection pressure in processes of language change (Karjus et al., 2020). Assessing our proposed neural network system against the discussion surrounding the FIT, we found that the TSC proved to be successful in that (a) it enabled us to efficiently, consistently, and accurately distinguish time series produced by stochastic drift from time series subject to selection pressure, while (b) solving a number of outstanding problems of the FIT (cf. Table 1). First, the TSC has only limited sensitivity to specific binning strategies. While the results of the FIT strongly depend on the number of temporal segments chosen, the predictions from the TSC are generally consistent regardless of the partitioning of the data. The difference in the impact of binning is especially prominent at higher selection coefficients. With fewer bins, the TSC's predictions remain accurate even with strong selection pressure. The performance of the FIT, on the other hand, degrades with increasingly fewer bins, erroneously flagging such cases as examples of stochastic drift. A second advantage of the TSC over the FIT is that the TSC does not make assumptions about specific distributions that underlie the data. For example, the FIT assumes that the frequency increments must be normally distributed, which is not a problem in itself, but facing distorted, real-world data, limits its application possibilities and reduces its statistical power.

**Table 1.** Overview of potential problems with detecting evolutionary forces in language change (and cultural change in general). Unsolved problems are marked as ×; problems solved with the time series classification task are marked as ✓. Problems in need of more research are marked as 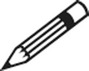.

Problem	Frequency Increment Test	Time series classifier
Variable number of bins	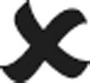	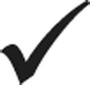
Variable selection strength	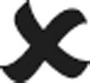	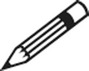
Non-normal frequency increments	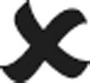	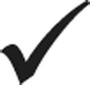
Distorted time series data	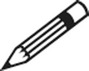	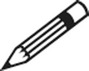
Entire change has not been observed	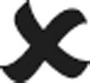	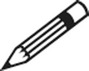

Thus, it appears that the neural TSC provides a solution to some of the major problems of the FIT. However, it must be acknowledged that this does not mean that the TSC solves all ‘99 problems’ of detecting evolutionary forces in language and cultural change. On the one hand, there are still several issues with the FIT that we have not addressed in the current study, and, on the other hand, the TSC itself is not without flaws either. We highlight two additional problems of the FIT mentioned in the literature (Karjus et al., 2020). First, the FIT assumes a constant selection coefficient *β* for the entire investigated period. However, it is not unlikely that *β* is unstable, and fluctuates over time. Second, the FIT struggles with incomplete time series, in which the entire process of change has not been observed. Whether the performance of the TSC is also affected by these issues cannot be ruled out without proper testing in the future. Yet the prospects are hopeful, as tackling such issues can be addressed by manipulating the artificially generated training material. An important advantage of the machine learning approach is the flexibility with which we can generate new training material. This allows us to prepare the system for incomplete time series, or series with variable selection strength.

At the same time, this inherent flexibility can unfortunately also be seen as a disadvantage of the supervised machine learning approach: after all, how can we make sure our simulated data is representative of real-world time series? Strictly speaking, this problem also applies to the FIT, as the motivation for its underlying *t*-test lies in the zero-mean frequency increments produced by the Wright–Fisher model. As a counterargument to such criticism, we thus wish to argue that, with a machine learning approach like the TSC, the problem becomes more explicit and imminent, thus forcing us to more thoroughly investigate how simulated data can be made more diverse and realistic. The data augmentation techniques applied in this article can serve as a first step, but future research should be directed toward investigating more extensive and comprehensive time series augmentation strategies (Fawaz et al., 2018).

Finally, we would briefly like to discuss the way we conceptualized the task of detecting evolutionary processes, that is, as a binary classification task. While this conceptualization makes the task efficient and simple, such binary all-or-nothing conceptualizations are not always the most informative. Consider, for instance, the information loss the binary approach entails in cases where the selection pressure is small (for example, <0.001): in such cases, it is more informative to know selection pressure exists in a small or negligible form (rather than simply lumping the case with all other cases of confirmed selection). In other words, instead of approaching questions of language evolution by classifying the time series into two categories, we may benefit more from an approach where we *infer* the selection pressure parameter from the data (see, for example, Newberry et al., 2017). In recent years, various methods and techniques have been developed to infer parameters based on simulation models (e.g. Crema et al., 2014; Kandler & Powell, 2015). Because the likelihood (the probability density for a given observation) is often intractable in complex simulation models, solutions are sought that bypass the computation of the likelihood. These so-called likelihood-free inference techniques – or, more generally, simulation-based inference techniques (Cranmer et al., 2019) – have been the focus of attention in recent years and have also been applied (with varying success) to cultural phenomena (Carrignon et al., 2019; Crema et al., 2014, 2016; Kandler et al., 2017; Kandler & Powell, 2015; Rubio-Campillo, 2016; Scanlon et al., 2019). One of the major stumbling blocks to these inference techniques is the curse of dimensionality and the consequential use of summary statistics, which reduce complex, multidimensional observations to a low-dimensional space. Crucially, the quality of the inference depends on whether the statistics are able to *sufficiently* summarize the observations, but it is often unclear which statistics are capable of doing so. A promising solution to this problem, again, can be found in machine learning algorithms (and in particular, neural networks), which allow us to work with high(er)-dimensional representations of the data, and thus circumvent the problem of summary statistics (Cranmer et al., 2015, 2019; Dinev & Gutmann, 2018; Gutmann et al., 2018; Hermans et al., 2019; Papamakarios et al., 2018). One such technique is the application of networks inspired by generative adversarial networks, which are trained to discriminate between data generated by parameter point *θ*_0_ from data simulated with *θ*_1_ (Cranmer et al., 2019; Hermans et al., 2019). We consider it a fruitful and exciting future line of research to investigate whether these new neural inference techniques can be combined with the neural network of the TSC, in order to improve the detection of – and, by extension, our understanding of – evolutionary forces in language and cultural change.

## Data Availability

Code to replicate the data used in this study can be downloaded from https://github.com/mnewberry/ldrift. All code and models to replicate the findings of the current study are available from https://github.com/fbkarsdorp/nnfit. Supplementary Materials with additional details about the neural networks, model training and data generation procedure are available from https://doi.org/10.5281/zenodo.4061776.

## Supplementary material

For supplementary material accompanying this paper visit http://dx.doi.org/10.1017/ehs.2020.52. click here to view supplementary material
