# Supplementary material for: Classifying evolutionary forces in language change using neural networks
Source: Evol Hum Sci. 2020 Oct 16;2:e50. doi: 10.1017/ehs.2020.52 (PMC10427472; doi:10.1017/ehs.2020.52)
Supplement: Supplementary file 1 [file S2513843X20000523sup001.pdf]

Supplementary Materials of "Classifying Evolutionary Forces in Language Change Using Neural Networks"

Folgert Karsdorp[1], Enrique Manjavacas[2], Lauren Fonteyn[3], and Mike Kestemont[2]

[1]Royal Netherlands Academy of Arts and Sciences, Meertens Institute, Amsterdam, The Netherlands

[2]Department of Literature, University of Antwerp, Antwerp, Belgium

[3]Leiden University Centre for Linguistics, Leiden University, Leiden, The Netherlands

October 5, 2020

1 Introduction

This document supplements the paper "Classifying Evolutionary Forces in Language Change Using Neural Networks". The document provides extensive details about the architecture of the neural networks, the generation of training data, the training procedure, and the parameters used to train the machine learning models. Additionally, we have supplemented all steps with Python code blocks to increase conceptual and technical understanding of the procedure, thus enabling other researchers to reproduce our results more easily as well as to apply the approach to their own work.

2 A Machine Learning Approach to Time Series Classification

In the paper, we conceptualize the task of detecting evolutionary forces in language and cultural change as a binary time series classification (TSC) task. To this end, we resort to a sequence classifier which will map an input in the form of a time series to one of two category labels (i.e. `0` for the absence and `1` for the presence of selection pressure in a time series). Formally, given a training data set D consisting of n pairs of time series X and their corresponding label vector $y \in 0, 1$, i.e., $D = (X_1, y_1), (X_2, y_2), \ldots, (X_n, y_n)$, the task of TSC is to learn a mapping function $f(X)$ for the input series to the output labels. By convention, we assume $y_i = 1$ when X_i was produced under selection forces, and $y_i = 0$ otherwise. Throughout, we will refer to the ground truth labels in the training data as y and refer to the predicted labels as $\hat{y}$.

Because we work with univariate time series, X will be a two-dimensional matrix of dimensionality (*number of time series* x *number of timesteps*); that of $\hat{y}$ will be (*number of time series* x *number of classes*). Note that the present work considers a binary, one-class categorization task, which explains why $\hat{y}$ is a one-dimensional vector (instead of a matrix). The network function f is controlled (or "parametrized") by a set of free parameters (Θ) that can be updated in the light of a given loss criterion or objective function that has to be minimized (see below).

2.1 Neural networks

The approach presented in the paper employs a Residual neural network (He et al. 2016), which has been successfully applied in the context of time series classification (Fawaz et al. 2019; Wang et al. 2016). In this section, we explain the various building blocks of this architecture in more detail. These are all standard components that are well established in the literature and implemented in all of the dominant frameworks for neural network research.

2.2 The Multi-Layer Perceptron

Before we describe the details of the Residual Networks, it might serve readers less familiar with the neural network literature to first briefly discuss a simpler network, the perceptron, and its extension, the multi-layer perceptron. The perceptron uses a single, linear projection to map the input matrix X straight onto $\hat{y}$. The perceptron is parameterized by Θ, i.e. a weight matrix (W) and a bias vector (b) associated with the affine projection of the input: $\hat{y} = f_\theta(X) = WX + b$. The dimensionality of W and b must be defined so that they are compatible with X. The **multi-layer perceptron** is an intuitive extension of the simple perceptron, where a series of n perceptrons or "layers" $(< l_1, l_2, ..., l_{n-1}, l_n >)$ are stacked on top of one another. Each layer (l_i) will pass on its own output as the input for the next perceptron (l_{i+1}) in a feedforward fashion, until finally, the output layer has been reached. In such cases, the function f can be rewritten as: $f(X) = (l_n(l_{n-1}(\ldots l_2(l_1(X))))$. The intermediate layers between the input and output in such a network are commonly called "hidden" layers. Mathematically, this model corresponds to a series of functions that are iteratively wrapped inside one another.

Each of these hidden layers is a discrete unit that can represent its own series of mathematical operations (as long as the resulting function is differentiable). Consequently, each layer is also parameterized by its own set of parameters $\Theta_i \in < \Theta_0, \Theta_1, ..., \Theta_{n-1}, \Theta_n >$. Inside a single layer, the basic projection $WX + b$ is moreover often followed by a (non-linear) **activation function**, such as the sigmoid function, to normalize the output into a more suitable range or distribution. Another widely used nonlinearity, also applied in the current paper, is the rectified linear unit (ReLU), a element-wise activation function that sets all negative values in a given matrix L to zero (i.e. $max(0, L)$). Finally, many layers implement a procedure known as "Batch Normalisation", which primarily functions as a regularization method.

2.3 Skip connections

The neural network architecture adopted in the paper makes use of **residual connections** (He et al. 2016): these extend the simple feedforward architecture, in which a given layer l_i can only directly feed the next layer (l_{i+1}). Residual networks keep the base mechanism of feedforward networks, but additionally allow for what is known as 'skip connections' between layers. This implies that a layer l_i can directly influence the input to layer $l_{i>i+1}$, thus effectively bypassing a number of layers in the stack. There exist several mechanisms to implement this concept. Consider a residual block of 4 hidden layers $(< l_1, ..., l_4 >)$ with the same dimensionality, in which we let l_1 skip two layers and directly affect the input to l_4. Then, a possible implementation would be: $l_4(X) = l_4(l_1(x) + l_3(l_2(l_1(X))))$. Here, the output of l_1 is directly passed to l_2, which itself feeds into l_3, as normal. The output of l_3, however, is subsequently summed with the original, unaltered output of l_1 (via an element-wise addition), before the result is eventually passed on as the input to l_4. This flow ensures that lower-level information can play a role in higher-level regions in the networks (and thus, for instance, cancel out lingering "residual" information).

2.4 Convolutions

Convolutional layers consist of a bank of **filters** (LeCun et al. 1998), that each independently apply a linear projection to the input layer (and, consequently, each filter comes with its own set of parameters Θ). There are three key distinctions with respect to a conventional linear layer, however. First of all, convolutional filters have a **smaller receptive field**: they are applied to a smaller slice of the input vector (i.e. a small number of timesteps in the case of univariate time series). This means that they are apt at extracting **local features** from the input, but are not sensitive to larger-scale trends in the time series. Secondly, the dimensionality of the time series can be severely affected, because there are *multiple* filters in a convolutional layer, that each get applied to the consecutive local segments in the time series. The original univariate time series, then, effectively becomes a *multivariate* time series after the convolutional operation, with a dimensionality equal to the number of filters applied. Thirdly – and this is a crucial characteristic – the same filter will get iteratively applied across various segments in the time series. This process is known as **weight sharing**, which ensures that the convolutional feature extraction is **spatially invariant**: the main idea supporting this approach is that the exact position of a particular feature in the time series should not matter and should be detected wherever it appears.

How the input is convolved is determined by a collection of manually set hyperparameters, including the number of filters in the layer, the length of the receptive field and the 'hop length' or 'stride' (≥ 1) in between consecutive applications of a filter. Naturally, these parameters will also affect the changing dimensionality of the input as it is fed through the network. Typically, stacks of convolutions are construed in a pyramidal fashion, so that the number of effective time steps in the original series is gradually reduced in the network. In this way, stacks of convolutional layers can become increasingly sensitive to larger-scale trends in the original signal. Mathematically, we shall denote a convolutional layer using the operator ($\otimes$): $y = \mathbf{W} \otimes X + \mathbf{b}$. Nowadays, convolutions are very common in machine learning, but they constitute a complex topic that cannot be fully covered here. For details, we therefore refer the many handbooks in the field, such as (Goodfellow et al. 2016). More practical handbooks are available too (Chollet 2017; Raschka 2015).

2.5 Full architecture

The full architecture of the neural network used in this paper combines all of the components introduced above: it is a residual neural network consisting of a stack of 9 convolutional layers that are divided in three residual blocks. Each residual block $r_i, i \in 1,2,3$, then, is composed of three convolutional layers, $h_i, i \in 1,2,3$. The layers in each block have 64 filters of size 8, 128 of size 5 and 128 of size 3, respectively. They each perform the same three operations:

$$
\begin{aligned}
y &= \mathbf{W} \otimes X + \mathbf{b} \\
s &= \mathrm{BatchNorm}(y) \\
h &= \mathrm{ReLU}(s)
\end{aligned}
\tag{1}
$$

where $\otimes$ is the convolution operator in the first line, and the third line refers to the application of the ReLU activation function covered above. The second line refers to a batch normalization layer (Ioffe and Szegedy 2015): we will not cover this detail here, as it is a standard component of modern neural networks. We shall briefly note that this layer applies a normalization operation to the previous layer's parameters, to reduce any dramatic covariance shifts in the parameters during training, which increases the network's overall numerical stability.

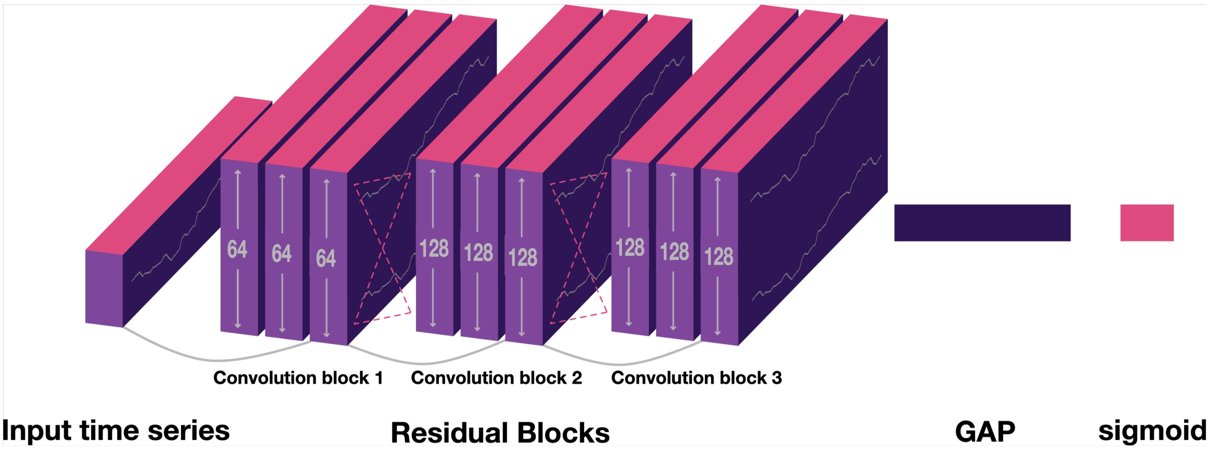

Figure 1: Schematic overview of the ResNet architecture. Each of the purple blocks represents a single layer; the convolutional layers are organized in residual blocks of length three. The numbers on the side of the boxes indicate the size of the filter bank in a convolutional layer. The grey bended curves indicate the "skip connections". After the convolutional part of the network, plain averaging (cf. GAP) is applied across the activation of the final convolutional layer to obtain a single output scalar for each instance. This number is finally bounded to the $[0, 1]$ range using the sigmoid function. The critical threshold for distinguishing the presence or absence of selection is set to 0.5.

The output matrix of the last residual block is transformed into a single scalar by averaging across the last layer's activations. This is also known as a 'Global Average Pooling' layer ('GAP'). This number is finally bounded to a pseudo-probability in the $[0, 1]$ range using the sigmoid function. Throughout, the critical threshold for distinguishing the presence or absence of selection adopted was 0.5.

The ResNet is implemented in Python using the Pytorch library (Paszke et al. 2019), and can be found in the file `src/nets.py` under the class `ResNet`:

```
%run ../src/nets.py
```

To initialize a Residual Network for our time series classification task, we set the number of input channels to 1, the number of filters of the first convolutional block to 64 (the remaining two are automatically set by multiplying the first by 2), and the number of output classes to 1, since we are dealing with a binary classification task:

```
model = ResNet(in_channels=1, mid_channels=64, num_pred_classes=1)
```

3 Generation of Training Data

3.1 Generating Time Series with the Wright-Fisher model

To effectively train the ResNet model, labeled training examples are required in order to optimize its weights (which are initialized randomly). To obtain this training material, we simulate artificial data using the Wright-Fisher model (Ewens 2012). In this section we describe and show how the training data has been generated. Using the Wright-Fisher model as described in Section 2.2.3 of the paper, we simulate time series representing frequency changes over time. The model assumes a population of constant size N and discrete, non-overlapping

generations. Let $z(t_i)$ be the number of times some cultural variant A occurs in generation t_i, and let $f(t_i)$ be the relative frequency of that variant. Equation (4) in the paper, then, provides the linear evolutionary pressure function used to generate the time series, which is repeated here for convenience:

$$g(f(t_i)) = \frac{(1+\beta)f(t_i)}{(1+\beta)f(t_i) + (1 - f(t_i))} \tag{4}$$

Here, β represents the bias towards selection of one of two variants of a cultural trait. The Wright-Fisher model is implemented in the Python function `wright_fisher`, which can be found in the file `src/simulation.py`. It can be used to generate time series with a variable number of generations (parameter `T`), different population sizes (parameter `N`), and varying selection coefficients (parameter `selection_strength`).

```
% run ../src/simulation.py
```

For example, to generate a time series based on a population size of $N = 1000$ for $T = 200$ timesteps without any selection pressure (i.e. $\beta = 0$), we can use the following lines of code:

```
neutral_series = wright_fisher(N=1000, T=200, start=0.5, selection_strength=0)
```

Similarly, to generate a time series with a positive selection pressure, we can write the following:

```
biased_series = wright_fisher(N=1000, T=200, start=0.5, selection_strength=0.02)
```

Using Python's plotting library Matplotlib (Hunter 2007), these series can be visualized as follows (cf. Figure 2):

```
import matplotlib.pyplot as plt
plt.style.use("bmh")

fig, ax = plt.subplots(figsize=(8, 5))

# normalize the series before plotting
ax.plot(neutral_series / 1000, label="neutral ($\\beta=0$)")
ax.plot(biased_series / 1000, label="biased ($\\beta=0.02$)")

ax.set(xlabel="$t$", ylabel="$f(t_i)$", ylim=(0, 1.1))
plt.legend(loc="upper center", ncol=2, bbox_to_anchor=(0.5, 1.1), frameon=False)
```

Note that the `wright_fisher` function employs a random number generator, resulting in different time series each time the cells above are executed. To illustrate this, consider the following two graphs in Figure 3, in which 100 time series with $\beta = 0$ and 100 with $\beta = 0.02$ are displayed. The code to generate these graphs is given by:

```
fig, (ax1, ax2) = plt.subplots(ncols=2, figsize=(10, 4), sharey=True)

for i in range(100):
    neutral_series = wright_fisher(N=1000, T=200, selection_strength=0) / 1000
    ax1.plot(neutral_series, alpha=0.1, color="C0", lw=1)

    biased_series = wright_fisher(N=1000, T=200, selection_strength=0.02) / 1000
```

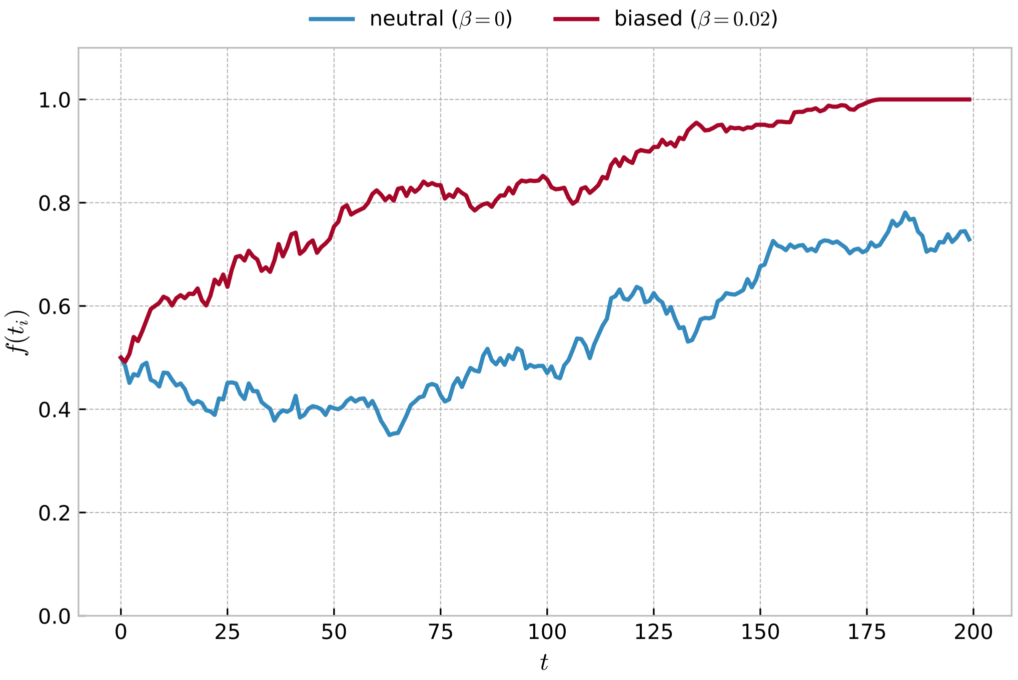

Figure 2: Time series of frequency changes generated with the Wright-Fisher model.

```
    ax2.plot(biased_series, alpha=0.1, color="C1", lw=1)

ax1.set(xlabel="$t$", ylabel="$f(t_i)$", ylim=(0, 1.1), title="$\\beta=0$")
ax2.set(xlabel="$t$", ylabel="$f(t_i)$", ylim=(0, 1.1), title="$\\beta=0.02$")

plt.tight_layout()
```

3.2 Data Distortion

In Section 2.2.4, the paper describes two data distortion strategies aimed at generating more realistic training samples and approximating real-world data aberrations. The first strategy is to distort the generated frequencies by augmenting them with an error term δ. For each time step $i = 1, 2, \ldots, T$, we sample δ from a normal distribution with zero mean and variance $\sigma = 0.2$:

$$f(t_i) = f(t_i) + \delta_i \qquad (2)$$
$$\delta_i \sim \text{Normal}(0, \sigma)$$

The augmented frequencies are subsequently truncated to the interval $[0, 1]$. To illustrate this strategy, consider the code block below and the resulting plot in Figure 4 in which we use the `Distorter` class (located in the file `src/utils.py` to alter the time series generated by the wright fisher model:

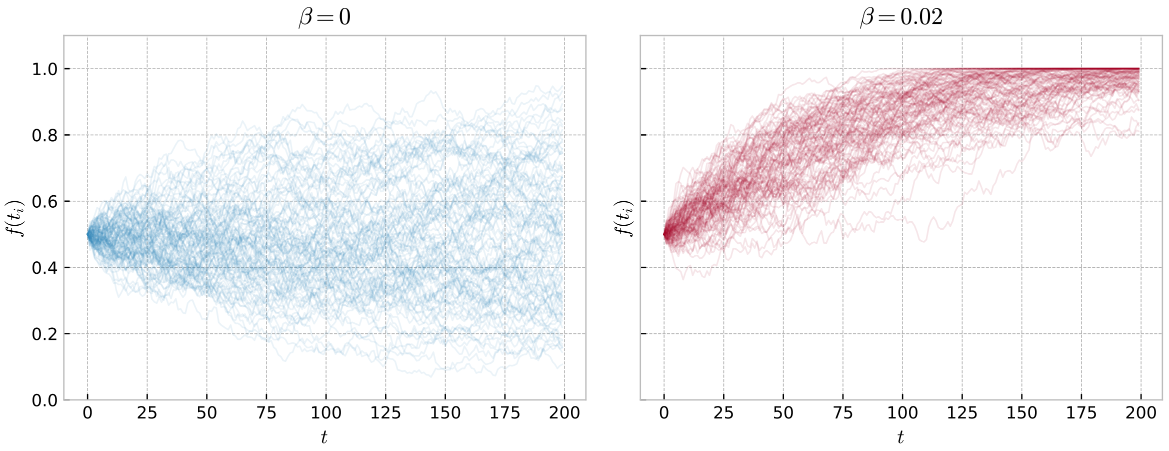

Figure 3: Time series generated with the Wright-Fisher model. The left panel displays 100 series generated with $\beta = 0$, and the right panel shows 100 time series with $\beta = 0.02$.

```
%run ../src/utils.py
```

```
disorter = Distorter(loc=0, sd=0.2)
series = wright_fisher(N=1000, T=200, selection_strength=0.02) / 1000
distorted_series = disorter.distort(series)

fig, ax = plt.subplots(figsize=(8, 5))

# normalize the series before plotting
ax.plot(series, label="undistorted", lw=1, zorder=4)
ax.plot(distorted_series, label="distorted", lw=1)

ax.set(xlabel="$t$", ylabel="$f(t_i)$", ylim=(0, 1.1))
plt.legend(loc="upper center", ncol=2, bbox_to_anchor=(0.5, 1.1), frameon=False)
```

The second strategy aims to mimic real-world time series distortions by grouping the time series into varying numbers of temporal segments ("bins"). Using this strategy, the generated counts are grouped into a number of bins (in the range $[4, T]$), and subsequently transformed into relative frequencies. A demo is given below of which the output is visualized in Figure 5:

```
series = wright_fisher(N=1000, T=200, selection_strength=0)
bins = 200, 50, 10, 4

fig, axes = plt.subplots(ncols=4, figsize=(10, 3), sharey=True)
axes = axes.flat

for i, n_bins in enumerate(bins):
    f = apply_binning(series, n_bins, n_agents=1000)
    axes[i].plot(f)
    axes[i].set(xlabel="$t$", title=f"$n$ bins = {n_bins}")
    if i == 0:
```

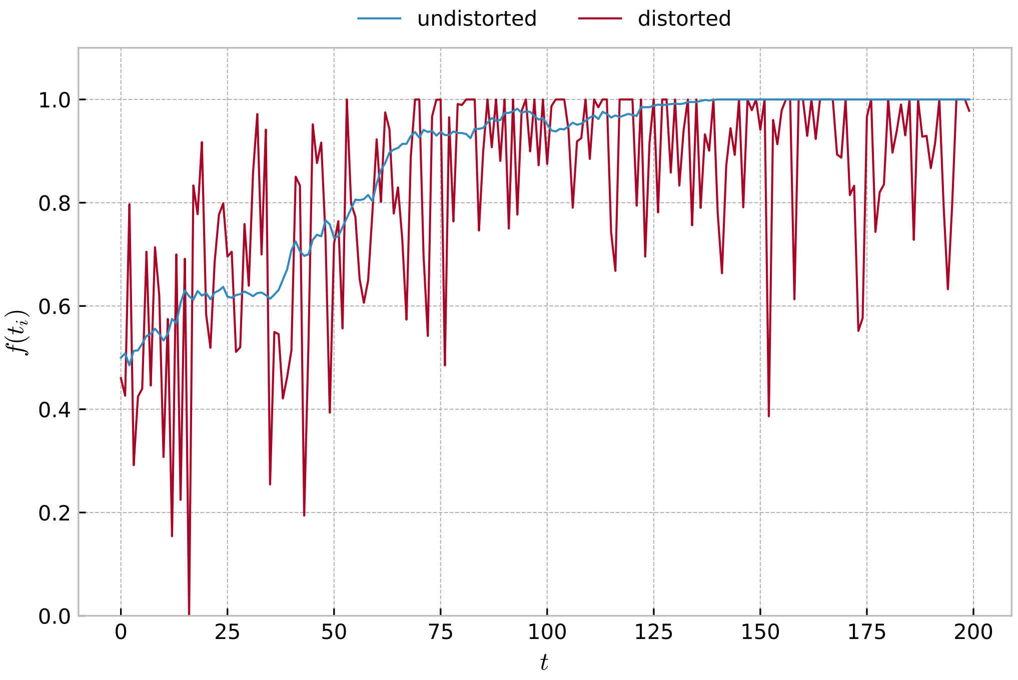

Figure 4: Time series generated with the Wright-Fisher model after following the described frequency distortion procedure.

```
        axes[i].set_ylabel("$f(t_i)$")

plt.tight_layout()
```

3.3 Generating batches of training data

The TSC is trained for a number of epochs, and at each epoch, n time series are generated. To efficiently train the neural classifier, we have to group these time series into mini-batches, which allows Pytorch to utilize more efficient matrix operations. Mini-batches are created using the class `SimulationBatch` located in the file `src/dataset.py`. This class conveniently wraps all time series generation functionality described above.

```
# When working on MacOS, uncomment the lines below, and run this unsafe cell
# This is needed for the DataLoader class described below, which uses multi-
# core processing for generating mini-batches

# import multiprocessing as mp
# mp.set_start_method("fork")
```

```
%run ../src/dataset.py
```

An instance of the batch sampler can be created as follows:

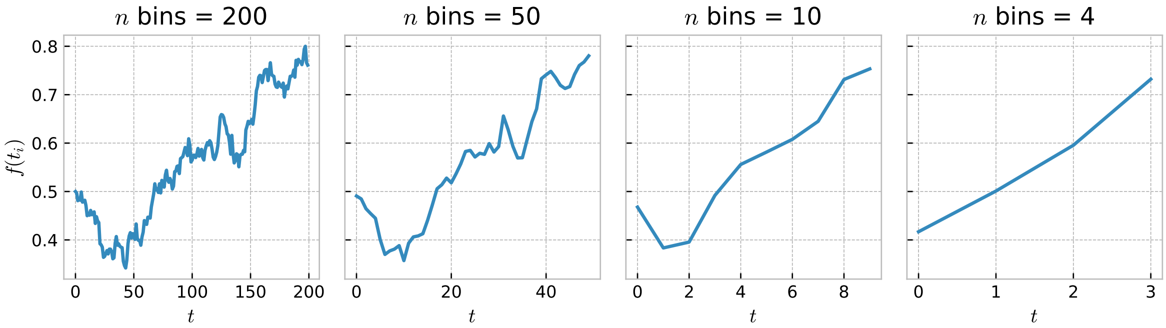

Figure 5: Time series after applying the binning strategy.

```
batch_sampler = SimulationBatch(
    n_agents=1000, timesteps=200, n_sims=1000, varying_start_value=True,
)
```

The class definition specifies a number of arguments, of which we will explain the most important ones. First, `n_agents` and `timesteps` specify the population size and number of timesteps respectively. Next, `n_sims` sets the number of samples generated in a mini-batch. Finally, by setting `varying_start_value` to `True`, each generated time series has a different starting frequency at $t_i = 0$, which is sampled from a uniform distribution $f(t_i) \sim \mathcal{U}(0.001, 0.999)$. When `False`, all samples start from the same given starting value.

As described in the paper, the batch sampler simulates each time series in a mini-batch using the `wright_fisher` function (see above) with a selection coefficient β in the range $[0, 1]$. In each mini-batch, a 50-50 split of positive ($\beta > 0$) and negative instances ($\beta = 0$) is generated. Positive selection coefficients are sampled from a log-uniform distribution (cf. Figure 6):

```
betas = loguniform(low=0.001, high=1, size=1000)

fig, ax = plt.subplots(figsize=(8, 5))
ax.hist(betas, bins="fd", density=True);
ax.set(xlabel="$\\beta$")
```

Subsequently, the generated time series are binned into a randomly sampled number of temporal segments and the bin values are distorted as described above. To generate a batch of time series, we call the sampler's `next()` method, which returns a tuple of (i) the labels Y, (ii) the sampled β values, (iii), the sampled number of bins for each time series, and (iv) the generated time series:

```
labels, beta, bins, series = batch_sampler.next()
```

The following lines confirm that the sampler made an equal split of positive and negative examples:

```
n_neutral, n_biased = (labels == 0).sum(), (labels == 1).sum()

print(f"number of neutral examples (beta = 0) : {n_neutral}")
print(f"number of biased examples (beta > 0):  {n_biased}")
```

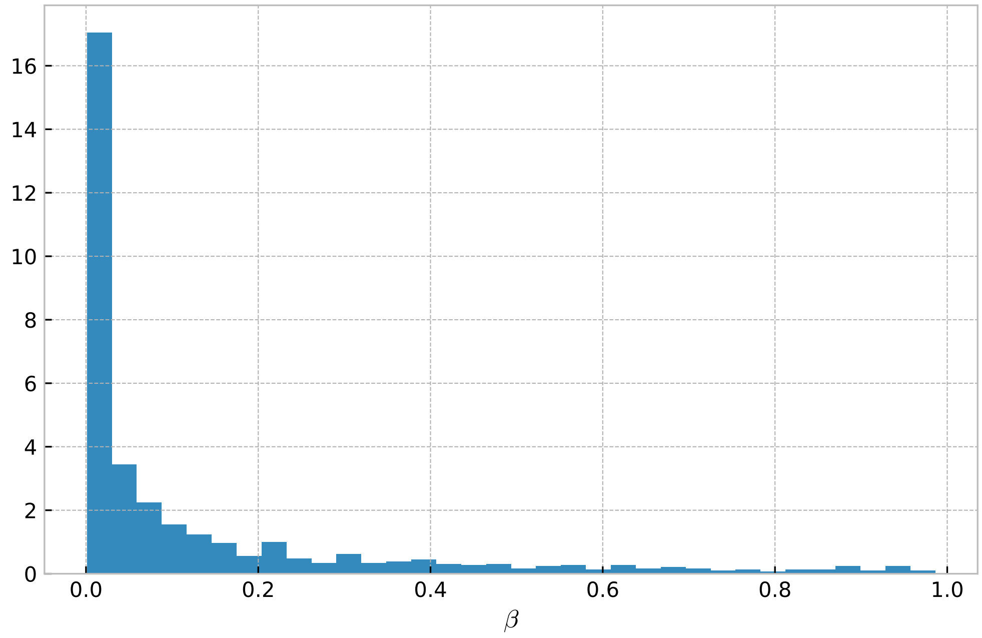

Figure 6: Distribution of β values sampled from the log-uniform distribution.

```
number of neutral examples (beta = 0) : 500
number of biased examples (beta > 0):  500
```

Similarly, the plots in Figure 7 show the distributions of the sampled values for β and number of bins:

```
fig, (ax_beta, ax_bins) = plt.subplots(ncols=2, figsize=(10, 4))

ax_beta.hist(beta.numpy(), bins="fd", density=True);
ax_beta.set(xlabel="sampled $\\beta$ value")

ax_bins.hist(bins.numpy(), bins="fd", density=True);
ax_bins.set(xlabel="sampled number of bins")

plt.tight_layout()
```

Finally, `series` as defined above contains a matrix of time series. To ensure that after varying the number of temporal segments, all time series in a mini-batch have the same length, zero-padding is applied to these series. This involves extending the series with zeros, as required by Pytorch:

```
tensor([[0.4450, 0.4505, 0.4860,  ..., 0.0000, 0.0000, 0.0000],
        [0.5849, 0.5410, 0.5686,  ..., 0.0000, 0.0000, 0.0000],
        [0.5906, 0.6934, 0.7471,  ..., 0.0000, 0.0000, 0.0000],
        ...,
        [0.4240, 0.4315, 0.4120,  ..., 0.0000, 0.0000, 0.0000],
```

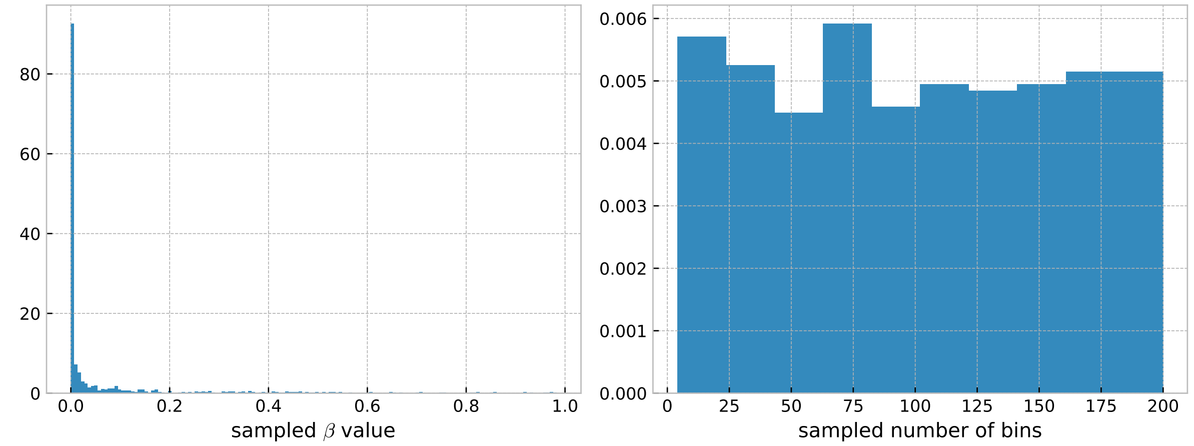

Figure 7: Distributions of sampled β values and number of temporal segments.

```
        [0.8958, 0.9991, 1.0000,  ..., 0.0000, 0.0000, 0.0000],
        [0.6665, 0.8210, 0.9215,  ..., 0.0000, 0.0000, 0.0000]])
```

```
print("Number of time series: {}, number of time steps: {}.".format(
    *series.size()))
```

```
Number of time series: 1000, number of time steps: 200.
```

4 Training Procedure

In this section, we will describe the training procedure outlined in the paper in more detail. To gain a more intuitive and conceptual understanding of the procedure, we supplement this description with lines of Python code to illustrate how the TSC can be trained.

4.1 A demonstration of the training procedure

The neural architecture of the TSC requires a large number of training examples to accurately and robustly discriminate between biased and unbiased series of frequency changes. To more efficiently generate a large number of mini-batches, the `SimulationBatch` class is wrapped in the class `DataLoader` (located in the file `src/datsets.py`), which enables generating mini-batches in parallel. The class requires a configuration dictionary which specifies the arguments for the `SimulationBatch` instances, as well as a batch size (`batch_size`) and number of simulations for each epoch (`n_sims`). Below we initialize a `DataLoader` instance, which will generate 1000 samples split into 1000 / 100 = 10 mini-batches:

```
config = {
    "n_agents": 1000,
    "timesteps": 200,
    "varying_start_value": True,
    "variable_binning": False,  # unused
```

```
    "start": 0.5,  # unused if variable_start_value = True
    "compute_fiv": False  # unused
}

data_loader = DataLoader(config, batch_size=100, n_sims=1000)
```

The cell below provides all necessary code to train the model. The model (see the variable `model` above) is trained for 10 consecutive epochs. In each epoch, the `DataLoader` generates a new set of time series. This set is fed to the ResNet model, and we aim to optimize its weights using the established Adam optimizer (Kingma and Ba 2015) with a small learning rate of 0.001. The loss function used is the binary cross-entropy loss (between y and $\hat{y}$), which we aim to minimize:

$$\ell(X,Y) = L = \frac{1}{N}\sum_{i=1}^{N} y_i \cdot \log(p(y_i)) + (1-y_i)\cdot \log(1-p(y_i)) \tag{3}$$

Here y_i is the binary label (1 or 0) for a particular time series, x_i, $p(y_i)$ the probability that x_i is generated with a non-zero selection coefficient, and N is the batch size.

```
import tqdm
import numpy as np
import torch
import torch.nn.functional as F

from torch.optim import Adam


learning_rate = 1e-3
n_epochs = 10
optimizer = Adam(model.parameters(), lr=learning_rate)
model.train()  # turn on training regime to allow parameter updates

train_loss = []
for epoch in tqdm.trange(n_epochs):
    epoch_loss = []
    for labels, _, _, series in data_loader:
        series = series.unsqueeze(1)
        labels = labels.unsqueeze(-1).float()
        # Set gradients to zero
        optimizer.zero_grad()
        outputs = model(series)
        # Compute the loss for this mini-batch
        loss = F.binary_cross_entropy_with_logits(outputs, labels)
        epoch_loss.append(loss.item())
        # Compute the gradient of loss for the model's parameters
        loss.backward()
        # Update the parameters Adam is supposed to update
        optimizer.step()
    train_loss.append(np.mean(epoch_loss))
```

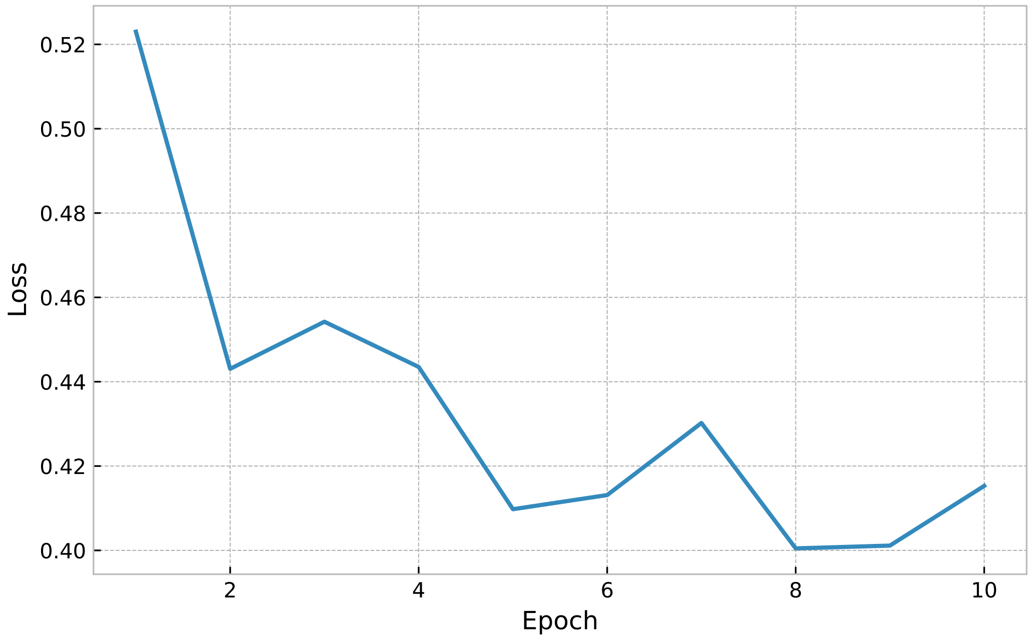


```
100\%|██████████| 10/10 [02:06<00:00, 12.63s/it]
```

Training the network for 10 epochs takes approximately 2 minutes on a 2,6 GHz 6-Core Intel Core i7 machine. The plot below visualizes how the loss progresses over time. It is assuring to observe that the loss is monotonically declining, even though the network is presented with new, unseen time series at each epoch. The declining loss indicates that the system is effectively learning how to discriminate between biased and unbiased samples better at each epoch:

```
fig, ax = plt.subplots(figsize=(8, 5))
ax.plot(np.arange(1, 11), train_loss)
ax.set(xlabel="Epoch", ylabel="Loss")
```

Note that the loss is still decreasing after 10 epochs and has not reached a plateau. Thus, with more epochs of training, the performance of the model could be improved (see below for a description of how the model described in the paper was trained). However, 10 epochs is enough for this demonstration of the model. Once the model is trained, we can use it to classify new, unseen time series as either "biased" or "unbiased" (i.e., "neutral"). For this classification we use the Sigmoid function, which returns the pseudo-probability p of a time series being biased. If $p > 0.5$, it is classified as biased, and unbiased otherwise (see the 'predict' function below).

```
def predict(series):
    """Helper function to make predictions about time series."""
    model.eval()  # Make sure no gradient updates take place
    with torch.no_grad():
        output = model(torch.FloatTensor([series]).unsqueeze(1))
        prob = torch.sigmoid(output).squeeze(1)[0]
```

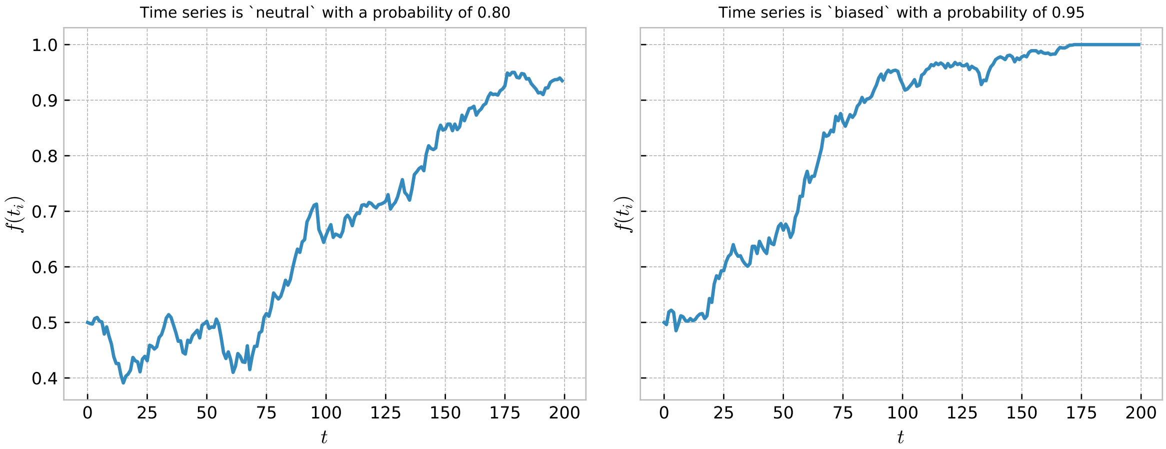

Figure 8: Time series and corresponding predictions of the TSC. The left panel shows a neutral time series (i.e. $\beta = 0$), and the right panel displays a series with biased frequency changes (i.e. $\beta = 0.02$). The titles of the two plot show the predictions of the classifier.

```
        pred = "biased" if prob > 0.5 else "neutral"
    if pred == "neutral":
        prob = 1 - prob
    return prob, pred
```

In the cell below, we test the model on two simulated time series. The first is a neutral time series ($\beta = 0$) and the second is generated with a low, but positive selection pressure ($\beta = 0.02$). Although the model has only been trained for a small number of epochs and on relatively few examples, it is already quite capable of correctly classifying the time series (cf. Figure 8):

```
neutral_series = wright_fisher(1000, 200, 0.0) / 1000
biased_series = wright_fisher(1000, 200, 0.02) / 1000

fig, (ax1, ax2) = plt.subplots(ncols=2, figsize=(10, 4), sharey=True)
ax1.plot(neutral_series)
ax1.set(xlabel="$t$", ylabel="$f(t_i)$")

prob, pred = predict(neutral_series)
ax1.set_title(f"Time series is `{pred}` with a probability of {prob:.2f}", fontsize=9);

ax2.plot(biased_series)
ax2.set(xlabel="$t$", ylabel="$f(t_i)$")

prob, pred = predict(biased_series)
ax2.set_title(f"Time series is `{pred}` with a probability of {prob:.2f}", fontsize=9);

plt.tight_layout()
```

4.2 Overview of the parameters used

In the demonstration above, we trained the TSC for 10 epochs. For better results, however, we should monitor the network's performance after each epoch on a held-out development set, and halt the training procedure after no improvement in the loss on the development data has been observed for five, consecutive epochs. The results described in the paper were obtained based on the following parameter settings:

1. We generated 50,000 time series per epoch, which are split into 100 mini-batches (the batch size is therefore 500).
2. The learning rate of the Adam optimizer was set to a low value of 6-e5 to prevent overfitting (although the exact value did not have much influence on the outcomes as long as it was less than 0.001).
3. The ResNet consists of three convolutional blocks that have 64 filters of size 8, 128 of size 5 and 128 of size 3.

It should be noted that the specific configuration generally only has a limited impact on the performance of the model, and that other neural architectures can yielded competitive performance (see the different models in `src/nets.py`). The most important point of the paper is that a machine learning approach, where the chosen classifier and architecture is of secondary importance, can achieve competitive performance, and possibly outperform other tests on real-world, messy data, that undermine particular assumptions of statistical tests.

References

Chollet, F. (2017). *Deep learning with python* (1st). USA, Manning Publications Co.

Ewens, W. J. (2012). *Mathematical population genetics 1: Theoretical introduction* (Vol. 27). Springer Science & Business Media.

Fawaz, H. I., Forestier, G., Weber, J., Idoumghar, L., & Muller, P.-A. (2019). Deep learning for time series classification: A review. *Data Mining and Knowledge Discovery*, *33*(4), 917–963.

Goodfellow, I., Bengio, Y., & Courville, A. (2016). *Deep learning* [http://www.deeplearningbook.org]. MIT Press.

He, K., Zhang, X., Ren, S., & Sun, J. (2016). Deep residual learning for image recognition, In *2016 IEEE conference on computer vision and pattern recognition, CVPR 2016, las vegas, nv, usa, june 27-30, 2016*, IEEE Computer Society.

Hunter, J. D. (2007). Matplotlib: A 2d graphics environment. *Computing in Science & Engineering*, *9*(3), 90–95.

Ioffe, S., & Szegedy, C. (2015). Batch normalization: Accelerating deep network training by reducing internal covariate shift (F. Bach & D. Blei, Eds.). In F. Bach & D. Blei (Eds.), Lille, France, PMLR.

Kingma, D. P., & Ba, J. L. (2015). Adam: A method for stochastic optimization, In *International conference on learning representations*.

LeCun, Y., Bottou, L., Bengio, Y., & Haffner, P. (1998). Gradient-based learning applied to document recognition, In *Proceedings of the ieee*.

Paszke, A., Gross, S., Massa, F., Lerer, A., Bradbury, J., Chanan, G., Killeen, T., Lin, Z., Gimelshein, N., Antiga, L., Desmaison, A., Kopf, A., Yang, E., DeVito, Z., Raison, M., Tejani, A., Chilamkurthy, S., Steiner, B., Fang, L., ... Chintala, S. (2019). Pytorch: An imperative style, high-performance deep learning library (H. Wallach, H. Larochelle, A. Beygelzimer, F. d'Alché-Buc, E. Fox, & R. Garnett, Eds.). In H. Wallach, H. Larochelle, A. Beygelzimer, F.

d'Alché-Buc, E. Fox, & R. Garnett (Eds.), *Advances in neural information processing systems 32*. Curran Associates, Inc.

Raschka, S. (2015). *Python machine learning*. Packt Publishing.

Wang, Z., Yan, W., & Oates, T. (2016). Time series classification from scratch with deep neural networks: A strong baseline. *arXiv preprint arXiv:1611.06455*.
